# 

**Published:** 1902-01-11

**Authors:** 


### NOTICE TO CORRESPONDENTS.

All MSS., Letters, Books for Review, and other matters intended for the Editor should be addressed The Editor, "The Hospital," The Hospital Building, 28 & 29 Southampton Street, Strand, W.C.

The Editor cannot undertake to return rejected MS., even when accompanied by stamped directed envelope.

**Notice to Advertisers and Subscribers.**

All Advertisements, Orders for Copies of The Hospital, and Business Communications should be addressed to **THE MANAGER (Not to the Editor),** The Hospital, 28 & 29 Southampton Street, Strand, London, W.C.

The Cost of Subscription, post free, is as follows:—Per week, 2½d.; per quarter, 2s. 8d.; per half-year, 5s. 3d.; per annum, 10s. 6d. Foreign:—Per annum, 15s. 2d., payable, in all cases, in advance.

**NOTICE.—Vol. XXX. of "The Hospital" (April 1901, to September 1901), in handsome cloth binding can now be obtained at the office of this Journal, price, 7s. 6d. Cases for binding the Numbers contained in this Volume 1s. 6d. Index to Vol. XXX., 2½d., post free.**

**The Monthly Part for January is now ready. Price 6d., by post 8d.**

### BOOKS RECEIVED.

J. and A. Churchill.

"A Short Practice of Midwifery for Nurses." By Henry Jellett, B.A., M.D., B.A.O. (Dublin Univ.), F.R.C.P.I., L.M.

"Nursing, General, Medical, and Surgical": with an Appendix on Sick-room Cookery. By Wilfrid J. Hadley, M.D., F.R.C.P. F.R.C.S.

P. Blakiston's, Son and Co., Philadelphia.

"A Pocket Cyclopædia of Medicine and Surgery." Edited by George M. Gould, A.M., M.D., and Walter L. Pyle, A.M., M.D.

### SCRAPS AND GLEANINGS.

THE Princess of Wales has sent a gift of pheasants to Queen Charlotte's Hospital, of which she is Vice-Patron.

H.R.H. PRINCESS CHRISTIAN has kindly consented to lay the foundation stone of the proposed Country Branch of the Mount Vernon Hospital for Consumption to be erected at Northwood, early next May. The Committee of the Hospital has received a donation of £500 from E. Steinkopff, Esq., towards the £3,000 still required to complete the hospital at Hampstead.

MESSRS. JAS. HENNESSY AND CO., of brandy fame, have been successful in obtaining an injunction restraining a trader from selling as their "Three Star" brandy a brandy which was not entitled to this description.

MESSRS. PARRISH'S Patent Steam-jacketed Cooker Company have received an order from the Government for jacketed pans and geysers to be sent to South Africa for cooking food for the mess.

THE Leathersellers' Company during 1901 made grants from their corporate funds amounting in all to £6,985, of which £3,161 was applied to hospitals and other charitable institutions and £1,466 to almspeople and pensioners.

THE December number of *The Studio* contained an interesting article describing Mr. George Cadbury's successful solution of the difficult problem of the housing of the working classes at the beautiful village of Bournville, near Birmingham.

THE British Home and Hospital for Incurables, Streatham, earnestly pleads for contributions to its funds. At present there are 78 inmates of the home and 344 pensioners, each receiving £20 a year. Altogether, on an average, the sum of £30 a day is given through the charity to helpless sufferers from all parts of the United Kingdom.

### THE STUDENT OF MEDICINE.

### APPOINTMENTS.

**W. Bannerman**, M.B., C.M.Edin., appointed Medical Officer for the Slaidburn District of the Clitheroe Union. **D. Brewer**, M.R.C.S.Eng., L.R.C.P.Lond., appointed an Assistant Medical Officer in the hospitals service of the Metropolitan Asylums Board. **Kenneth Campbell**, L.R.C.P., L.R.C.S.Edin., L.F.P.S.Glasg., appointed Medical Officer to the Parish Council of Kilmore and Kilbride. **J. Chronnell**, M.R.C.S.Eng., L.R.C.P.I., appointed Medical Officer of Health for the Hindley Urban District. **Harold Collinson**, M.B., M.R.C.S., L.R.C.P., appointed Resident Surgical Officer to the General Infirmary at Leeds. **David Ferrier**, M.B., Ch.B.Edin., appointed House Surgeon to the Salisbury Infirmary. **J. Graham Forbes**, M.A., M.D., D.P.H., appointed Medical Officer to the Gold Coast Anglo-French Boundary Commission, West Africa. **A. S. Gedge**, M.R.C.S., L.R.C.P.Lond., appointed Medical Officer for the Fifth District of the Devizes Union. **Kenneth W. Goadby**, L.D.S.Eng., D.P.H.Camb., appointed Bacteriologist and Lecturer on Bacteriology to the National Dental Hospital. **J. N. Hall**, M.R.C.S., L.R.C.P.Lond., appointed Medical Officer for the Mayfield District of the Uckfield Union;

**T. N. Kelynack**, M.D., M.R.C.P., appointed Assistant Physician to the Mount Vernon Hospital for Consumption and Diseases of the Chest. Hampstead and Northwood. **H. Lambert Lack**, M.D.Lond., F.R C.S., appointed Aural Surgeon to the London Hospital. **W. C. Moore**, M.B., B.C.Cam., appointed Medical Officer of Health for the Stow-on-the-Wold Urban District. **R. Niven**, M.B., B.Ch.Glasg., D.P.H.Camb., appointed an Assistant Medical Officer in the hospitals service of the Metropolitan Asylums Board. **J. A. Parsons**, M.D., appointed Medical Officer for the Clipsham District of the Stamford Union. **L. L. Preston**, M.B., B.S.Durh., appointed Medical Officer of Health for the St. Helen's Urban District, Isle of Wight. **J. A. Purdon**, L.R.C.P., L.R.C.S.I., appointed Medical Officer for the Pinchbeck District of the Spalding Union. **Wm. Redpath**, M.R.C.S.Eng., L.R.C.P., appointed Medical Officer of Health to the Woodbridge Urban District Council. **C. A. S. Ridout**, M.B., B S.Lond., M.R.C.S., L.R.C.P., appointed House Surgeon to the North Staffordshire General Infirmary and Eye Hospital, Stoke-on-Trent. **W. J. Shannon**, L.R.C.P., L.R.C.S.Edin., L.F.P.S.Glasg., appointed District Medical Officer of the Biggleswade Union. **Edwin Charles Temple Smith**, M.B, Ch.B., R.U.I., M.R.C.S.Eng., L.R.C.P.Lond., appointed an Honorary Surgeon to the General Hospital, and to be Medical Officer of the Children's Hospital, Charters Towers, Queensland. **Oswald Smith**, M.B., B.S.Edin., appointed House Physician to the Salisbury Infirmary. **A. R. Spencer**, M.B., M.R.C.S., L.R.C.P., appointed an Assistant Medical Officer in the hospitals service of the Metropolitan Asylums Board. **Thomas George Stevens**, M.D.Lond., F.R.C.S.Eng., M.R.C.P.Lond., appointed Obstetric Tutor to St. Mary's Hospital. **M. R. Taylor**, M.R.C S., L.R.C.P.Lond., appointed District Medical Officer of the Helston Union. **Reginald Charles Verley**, M.B., Ch.B.Edin., M.R.C.S.Eng., L.R.C.P.Lond., B.Sc., appointed Resident Medical Officer to the St. Marylebone Dispensary, Welbeck Street, W. **G. S. Ware**, M.B., B.S.Durh., appointed District Medical Officer of the Barnstaple Union. **W. H. T. Winter**, M.R.C.S., appointed Medical Referee under the Workmen's Compensation Acts for the Wolverhampton County Court District of Circuit No. 25.

